# Supplementary material for: Serum FHR1 binding to necrotic-type cells activates monocytic inflammasome and marks necrotic sites in vasculopathies
Source: Nat Commun. 2019 Jul 4;10:2961. doi: 10.1038/s41467-019-10766-0 (PMC6609651; doi:10.1038/s41467-019-10766-0)
Supplement: Supplementary file 1 — Supplementary Information [file 41467_2019_10766_MOESM1_ESM.pdf]

Supplementary Informations

Serum FHR1 binding to necrotic-type cells activates monocytic inflammasome and marks necrotic sites in vasculopathies

Irmscher *et al.* 2019

Supplementary Table 1: Overview of conditions and inhibitors

Supplementary Table 2: Overview of AAV patients

Supplementary Figure 1: FHR1 induced inflammation depends on immobilization and NHS

Supplementary Figure 2: FHR1 activates NLRP3 in monocytes via the PLC pathway

Supplementary Figure 3: Binding of FHR1 to necrotic-type cells and MDA-LDL

Supplementary Figure 4: Characteristics of AAV cohort

Supplementary Table 1. Overview of conditions and inhibitors

	Condition/reagent	Inhibited/not present	IL-1β induced by FHR1
**Surface**	FHR1 immobilized		+
	Soluble FHR1		-
	FHR1 bound to C3b		-
	FHR1 on necrotic cells		+
	FHR1 bound to MDA-LDL		+
**NHS**	Active NHS		+
	No NHS	NHS	-
	EDTA NHS	NHS	-
	Heat-inactivated NHS	NHS	-
**Complement system** (CP = classical-, LP = lectin-, AP = alternative pathway)	C3-depleted NHS	C3	+
	Compstatin	C3	+
	Eculizumab	C5	+
	EGTA	CP+LP	+
	C1q-depleted NHS	CP	+
	FB-depleted NHS	AP	+
	FP-depleted NHS	AP	+
**Intracellular**	BAY-117085	NFkB	-
	VX765	Caspase 1	-
	MCC950	NLRP3	-
	U73122	PLC	-
	Phenantroline	PLC, metallo prot.	-
	No ROS release detectable	ROS	+
	Glybenclamide	K^+efflux	+
	Cathepsin B inhibitor	Cathepsin B	+
**Receptors**	Simvastatin	CR3	+
	TAS	C3aR	+
	α-TLR2	TLR2	+
	LPS-RS	TLR4	+
	α-TLR6	TLR6	+
	α-CD14	CD14	+
	α-CD36	CD36	+
	WGP	Dectin1	+
	FcRblocker	FcR	+
	α-RAGE	Human RAGE	+
	Gallein	Gβγ of GPCRs	-
	αEMR2	EMR2	-

Supplementary Table 2. Overview of AAV patients

Disease	FHR1/3	n (%)	Male n (%)	Female n (%)	Age (Ø)	IL-1β pg mL^{-1} (Ø)	CRP mg dL^{-1} (Ø)
**AAV**	total	314					
	FHR1/3	303 (96.5)	206 (68)	97 (32)	18-90 (63)	0-65(1.4)	5-194.6 (36.9)
	ΔFHR1/3	11 (3.5)	7 (63.6)	4 (36.4)	39-83 (61)	0.1 -1.2 (0.6)	5-7.8 (5.4)
**Ctrl.**	total	55	n.d.	n.d.	n.d.		
	FHR1/3	51 (92.7)				0-3.1 (0.4)	5
	ΔFHR1/3	4 (7.3)				0-0.6 (0.2)	5

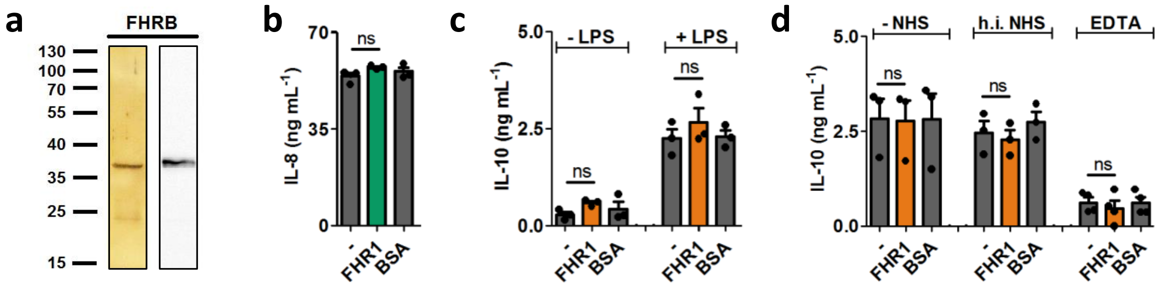

Supplementary Figure 1

FHR1 induced inflammation depends on immobilization and NHS

a, Recombinant expression of murine homolog of FHR1, FHRB, is shown by silvergel (left) and western blot (right). **b,** FHR1 does not affect IL-8 secretion. **c,** Unbound FHR1 fails to reduce IL-10 secretion. **d,** In the absence of NHS, and upon exposure to heat-inactivated (h.i.) and EDTA-inactivated NHS, immobilized FHR1 does not inhibit IL-10 secretion by LPS-stimulated monocytes. Data in a-d represent the mean ± SEM. of three to four independent experiments using cells from different donors (unpaired two-tailed t-test).

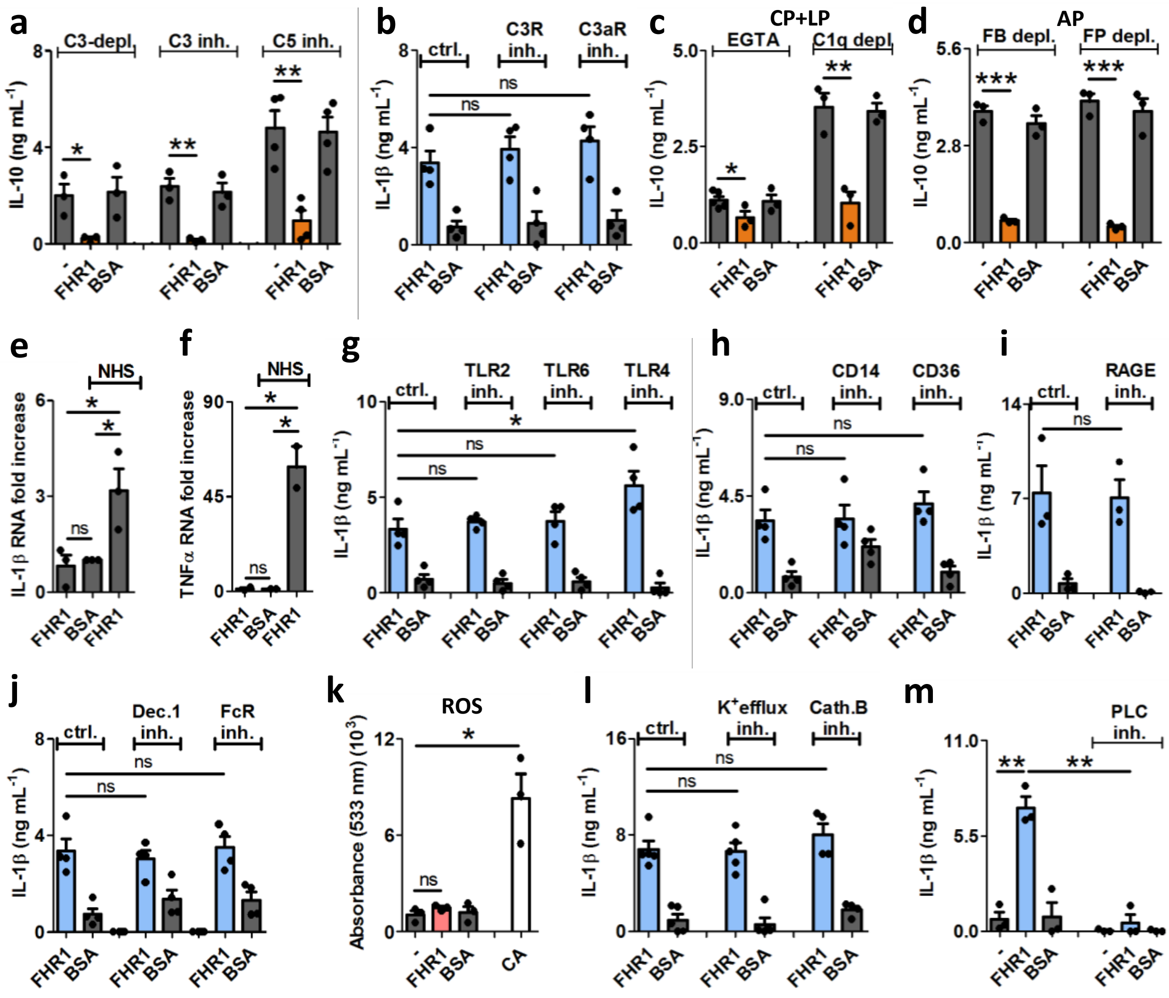

Supplementary Figure 2

FHR1 activates NLRP3 in monocytes via the PLC pathway.

a, Immobilized FHR1 inhibits IL-10 release by monocytes exposed to C3-depleted, C3-inhibited (Compstatin), or C5-inhibited (Eculizumab) NHS. **b,** Blocking of CR3 with simvastatin or C3aR with TAS does not reduce FHR1-induced release of IL-1β from monocytes when compared with that from untreated control (ctrl.) cells. **c,** FHR1 inhibits IL-10 secretion upon blockade of the classical (CP) and lectin pathways (LP) (EGTA-inhibited or C1q-depleted NHS), **d,** and upon blockade of the alternative pathway (AP) (NHS depleted of factor B (FB) or factor P (FP)). **e,** FHR1 increases the amount of IL-1β and **f,** TNFα mRNA in monocytes in the presence of NHS, but not in its absence (n=2). **g-j,** Blockade of TLR2 (by α-TLR2), TLR4 (by LPS-RS),

TLR6 (by α-TLR6), CD14 (by α-CD14), CD36 (by α-CD36), human RAGE (by α-RAGE), Dectin-1 (by WGP) and FcR (with a FcR blocker) does not reduce FHR1-induced secretion of IL-1β when compared with the control. **k,** FHR1 does not trigger ROS release in monocytes (NHS), but *C. albicans* (CA) does. **l,** FHR1 induces secretion of IL-1β in the presence of a K^+ efflux inhibitor (glybenclamide), cathepsin B inhibitor (Cath.B inh.), but not with **m,** PLC (phenanthroline) inhibitor. Data in a-m represent the mean ± SEM. of three to five independent experiments unless specified otherwise. *p≤0.05, **p≤0.01, ***p≤0.001 (unpaired two-tailed t-test).

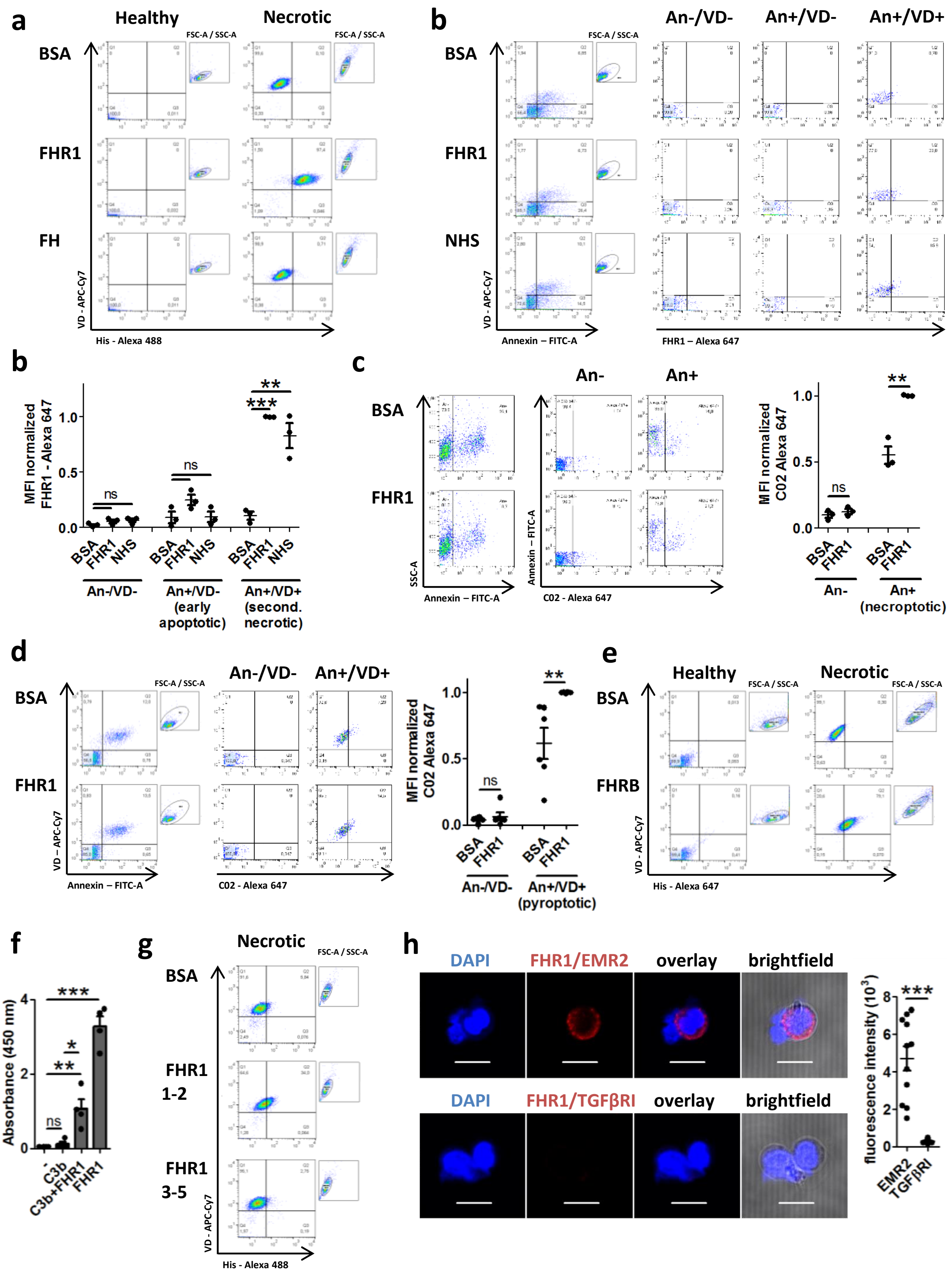
a
Healthy
Necrotic
BSA
FHR1
FH
VD - APC-Cy7
His - Alexa 488
b
An-/VD-
An+/VD-
An+/VD+
NHS
Annexin – FITC-A
FHR1 – Alexa 647
MFI normalized FHR1 - Alexa 647
ns
***
**
An-/VD-
An+/VD- (early apoptotic)
An+/VD+ (second. necrotic)
c
An-
An+
SSC-A
Annexin – FITC-A
C02 - Alexa 647
MFI normalized C02 Alexa 647
An+ (necroptotic)
d
An+/VD+ (pyroptotic)
e
FHRB
His - Alexa 647
f
Absorbance (450 nm)
C3b
C3b+FHR1
FHR1
g
FHR1 1-2
FHR1 3-5
h
DAPI
FHR1/EMR2
overlay
brightfield
FHR1/TGFβRI
fluorescence intensity (10^3)
EMR2
TGFβRI

Supplementary Figure 3

Binding of FHR1 to necrotic type cells and MDA-LDL.

a, FHR1, but not FH, binds necrotic Viability Dye (VD) positive HUVECs, as shown by flow cytometry using monoclonal C02 antibody which stains SCR19 of FH and SCR4 of FHR1. **b,** Binding of FHR1 and natural FHR1 (NHS) to necrotic HUVECs (An+/VD+), but not to early apoptotic (An+/VD-) or living (An-/VD-) cells using flow cytometry and monoclonal FHR1 antibodies. **c,** FHR1 binds to necroptotic U937 cells (An+), but not living cells (An-), using flow cytometry and monoclonal C02 antibodies. **d,** Binding of FHR1 to pyroptotic THP1 cells (An+/VD+), but not to double negative (An-/VD-) cells, using flow cytometry and monoclonal C02 antibodies. An = Annexin, VD = viability dye. ,**e,** FHRB binds to necrotic mouse alveolar macrophages (VD positive) but not to healthy cells, as shown by flow cytometry using FHRB antiserum. **f,** FHR1 binds to C3b as shown by ELISA. Data represent mean ± SEM. of four independent experiments. **g,** FHR1 SCR1–2 binds to necrotic HUVECs (VD positive), as shown by flow cytometry using a monoclonal His-Tag antibody. **h,** Complexes (red) of FHR1 (on necrotic cells) with EMR2 receptor (on monocytes) or TGFβRI in whole blood. Total picture fluorescence intensity of single cells (EMR2 n=11, TGFβRI n=17) were analyzed using unpaired two-tailed t-test, ***p≤0.001. Data in a-e and g show Dot Plots of one of at least three independent experiments. *p≤0.05, **p≤0.01, ***p≤0.001 (unpaired two-tailed t-test).

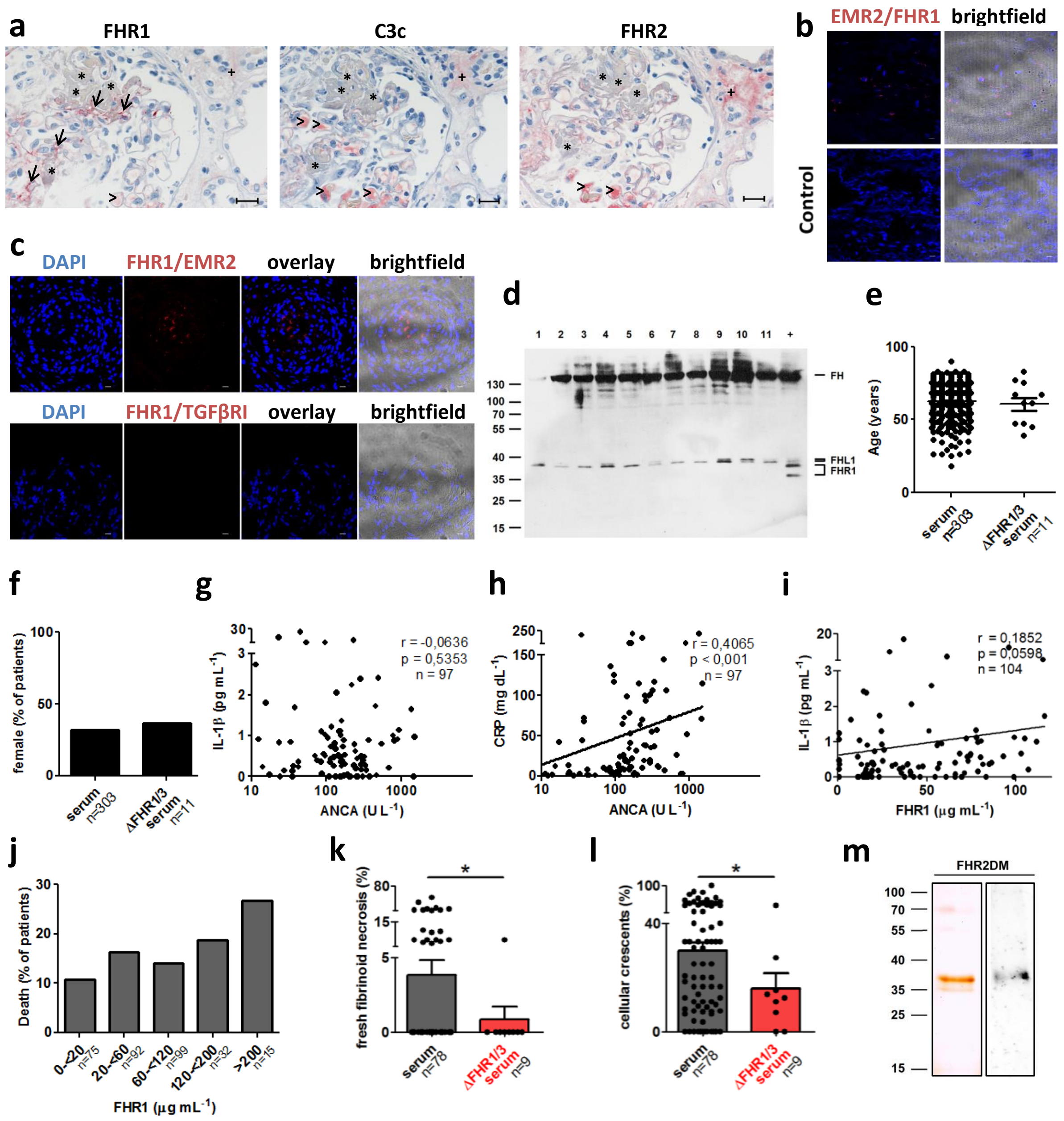

Supplementary Figure 4

Characteristics of AAV cohort.

a, FHR1, C3c and FHR2 staining in AAV kidney biopsies: Granular signals for FHR1 (arrows, left panel) in parts of the glomerular matrix that are in proximity to fibrinoid necrosis („*" = fibrin precipitates). In contrast, almost no staining signals of C3c (middle panel) and FHR2 (right panel) is almost negative in these areas (same glomerulus). „>" marks likely uncharacteristic serum signals in the glomerular

capillaries and „+“ marks (possibly uncharacteristic) signals in the tubulointerstitium. Bars = 20 µm. **b,** EMR2/FHR1 complexes in atherosclerotic plaques (PLA). DNA is stained by DAPI. Control = one antibody. **c,** EMR2/FHR1 complexes, but no TGFβRI/FHR1 complex formed in kidney biopsies of AAV patients shown by PLA assay. DNA is stained with DAPI. Bar = 10 µm. **d,** Eleven AAV patients (11/314) lack FHR1 and FHR3 (not shown) as seen by Western Blot analysis. NHS with FHR1 is marked with (+). **e,** AAV patients with FHR1/3 or ΔFHR1/3 are of similar age (mean ± SEM) and **f,** gender (mean). **g,** Levels of ANCA antibodies in AAV patients do not correlate with IL-1β concentrations, but with **h,** CRP amounts in the samples. **i,** Correlation between IL-1β and FHR1 concentrations in AAV patients without immunosuppressive therapy (g-i Spearman correlation). **j,** The percentage of deaths of AAV patients increase with FHR1 concentrations. **k,** Fresh fibrinoid necrosis and **l,** cellular crescents of AAV with FHR1/3 compared to ΔFHR1/3 AAV patients (unpaired two tailed t-test, Welch’s correction). **m,** Recombinant expression of dimerization mutant FHR2DM (silver gel (left) and western blot (right)). One representative gel out of three is shown.
